# Pathways for non-manufacturers to drive generic drug repurposing for cancer in the U.S.

**DOI:** 10.3389/fphar.2024.1419772

**Published:** 2024-10-09

**Authors:** Devon Crittenden, Raquel Gallagher, Fernanda Milans del Bosch, David M. Fox, Laura B. Kleiman

**Affiliations:** Reboot Rx Inc, Boston, MA, United States

**Keywords:** drug repurposing, oncology, policy, off-label use, FDA approval process, regulatory framework, cancer

## Abstract

Repurposing generic drugs as new treatments for life-threatening diseases such as cancer is an exciting yet largely overlooked opportunity due to a lack of market-driven incentives. Nonprofit organizations and other non-manufacturers have been ramping up efforts to repurpose widely available generic drugs and rapidly expand affordable treatment options for patients. However, these non-manufacturers find it difficult to obtain regulatory approval in the U.S. Without a straightforward path for approval and updating drug labeling, non-manufacturers have relied on off-label use of repurposed drugs. This limits the broad clinical adoption of these drugs and patient access. In this paper, we explore the regulatory landscape for repurposing of small molecule generic drugs within the U.S. We describe case studies of repurposed drugs that have been successfully incorporated into clinical treatment guidelines for cancer without regulatory approval. To encourage greater adoption of generic drugs in clinical practice–that is, to encourage the repurposing of these drugs–we examine existing Food and Drug Administration (FDA) pathways for approval of new uses or indications for generic drugs. We show how non-manufacturers, who are generally more active in generic drug repurposing than manufacturers, could utilize existing regulatory authorities and pathways, and we describe the challenges they face. We propose an extension of the existing 505(b)(2) new drug application (NDA) approval pathway, called a “labeling-only” 505(b)(2) NDA, that would enable non-manufacturers to seek approval of new indications for well-established small molecule drugs when multiple generic products are already available. It would not require new chemistry, manufacturing, and controls (CMC) data or introducing new drug products into the marketplace. This pathway would unlock innovation broadly and enable patients to benefit from the enormous potential of low-cost generic drugs.

## Introduction: The opportunity for generic drug repurposing in oncology

Each year, 10 million people worldwide die of cancer (Sung et al., 2021). Cancer patients urgently need more effective and affordable treatment options, yet developing one new drug can take over a decade and $1 billion (Prasad and Mailankody, 2017). The pathway from initial drug discovery to regulatory approval spans from analytical and preclinical testing to multiple phases of clinical trials and rigorous safety assessments, all of which drive up the time and cost of drug development (Lo et al., 2009). Once new cancer drugs reach the market, they often extend survival by just a few months and cost over $100,000 annually (Prasad et al., 2017; Gyawali et al., 2019; Michaeli and Michaeli, 2022). Due to the high cost of care, around 40% of cancer patients in the U.S. exhaust their life savings within 2 years of diagnosis (Gilligan et al., 2018).

Generic drug repurposing aims to identify and implement new therapeutic uses for approved and off-patent drugs, and thereby streamlines the drug development process by leveraging existing clinical and safety data (Kulkarni et al., 2023; van der Pol et al., 2023; Weth et al., 2023). Repurposing opportunities are discovered in various ways, such as preclinical screens and serendipitous clinical observations (Bertolini et al., 2015; Orecchioni et al., 2019). Generics are generally well-established drug compounds with known drug compositions and safety profiles based on years of clinical use (van der Pol et al., 2023; Weth et al., 2023).

Generic drug repurposing is not being realized to its full potential due to a lack of financial incentives (Bertolini et al., 2015). Pharmaceutical companies are primarily focused on *de novo* drug development to create new molecular and chemical entities (Congressional Budget Office, 2021). Once drug patents and marketing exclusivities expire, the drugs can be manufactured and sold by any company, subject to obtaining regulatory approval. With the increased supply, competition in the marketplace causes prices to drop significantly; generics can be up to 80%–85% less expensive than branded versions (U.S Food and Drug Administration, 2023a; U.S. Food and Drug Administration, 2023b). The low profit margins for generic drugs mean that pharmaceutical companies no longer want to invest in research and marketing for new uses (Juárez-López and Schcolnik-Cabrera, 2021).

Pharmaceutical companies increasingly lose interest in obtaining approval for new indications of drugs the closer they are to generic entry (Sahragardjoonegani et al., 2021; Liddicoat et al., 2022). Typically, they are only interested in repurposing while the drugs are still protected by patents and marketing exclusivities, or if they can change the drugs in some way to create new patent protection, such as through new formulations, dosages, or routes of administration (Langedijk et al., 2016; Beall et al., 2019; Sahragardjoonegani et al., 2021). For example, the vitamin A derivative all-trans retinoic acid (ATRA) was reformulated from a topical cream for treating acne (*Retin-A*) into an oral capsule for treating acute promyelocytic leukemia (*Vesanoid*) (Integrated Therapeutic Solutions, 1995; Yoham and Casadesus, 2023). The new oral formulation provided marketing exclusivity for the promotion of the new product for the new indication (U.S. Food and Drug Administration, 1990).

There are many opportunities to repurpose generic drugs without making any changes to the existing, approved versions (Weth et al., 2023). During the development of new molecular and chemical entities, and in some cases after regulatory approval and before patent expiration, pharmaceutical companies may explore multiple indications and only pursue a small fraction of them (Mills et al., 2023). Academics funded by governments and disease foundations also conduct research into new indications (Verbaanderd et al., 2020; del Álamo et al., 2022). In oncology, many generic drugs approved for non-cancer uses have been tested as cancer treatments; published preclinical and early clinical studies demonstrate that more than 300 non-cancer generics have anticancer potential (Pantziarka et al., 2018). Yet there is a lack of funding for practice-changing clinical trials evaluating new indications for generic drugs, which is a challenge widely discussed in the field (Pushpakom et al., 2019; van der Pol et al., 2023).

Nonprofit organizations and other “non-manufacturers” are now emerging to advance generic drug repurposing research (Naylor et al., 2015; Pantziarka et al., 2021; Weth et al., 2023). Non-manufacturers, which do not manufacture or distribute drugs, face impediments in pursuing regulatory approval for new indications, which is limiting the impact for patients. While new indications can be prescribed off-label, regulatory approval and updated labeling would offer significant advantages for driving widespread adoption, meaning the maximum usage of the drugs in clinical practice (Wittich et al., 2012). In this paper, we review the challenges non-manufacturers face in pursuing regulatory approval and propose a viable solution.

## Non-manufacturers are advancing generic drug repurposing

Pharmaceutical companies could repurpose generics without changing the drugs and apply for method-of-use patents, which in theory should provide exclusivity for new indications and the potential for higher pricing (Gupta et al., 2010; Roin, 2013; Rai and Rice, 2014). However, due to substitution of generic drugs at the pharmacy level, method-of-use patents are of little to no practical value when there are already therapeutically equivalent products on the market (Breckenridge and Jacob, 2019). Pharmacists can dispense any equivalent generic versions instead of the patent-protected drug products, even if the substituted generic does not have the specific indication in its labeling (Rome et al., 2022; Walsh et al., 2022). Currently, over a third of U.S. states have regulations that require generic substitution when available (Song and Barthold, 2018; Sacks et al., 2021).

In some cases, no additional research may be needed to support the use of repurposed generics, yet pharmaceutical companies and generic drug manufacturers are still unlikely to pursue regulatory approval for new labeling and promote the new indications. There is no legal obligation for them to seek approval, and it may not be risk proportionate for them, given financial considerations, potential liabilities, and associated responsibilities (U.S. Food and Drug Administration, 2013; Shea et al., 2018; van der Pol et al., 2023).

To address this market failure, some governments have created publicly funded programs to facilitate regulatory approval, such as:

1. *Project Renewal through the U.S. Food and Drug Administration (FDA) Oncology Center of Excellence.* Project Renewal is supporting updates to the labeling of certain older oncology drugs where the labeling is outdated and does not reflect their current clinical uses (Kluetz et al., 2021). The newer oncology indications may be well recognized in the medical community and supported by published reports of adequate clinical data (Oncology Center of Excellence, 2023b). Project Renewal gathers the supportive evidence and then engages the pharmaceutical company that developed the branded product to amend the labeling (Kluetz et al., 2021). The initial focus of Project Renewal is on long-standing drugs whose oncology indications have been the standard of care for years; newly repurposed treatments are not currently included within its scope (Oncology Center of Excellence, 2023b).
2. *The Medicines Repurposing programme led by the National Health Service (NHS) in England.* For select drugs, once there is sufficient evidence of safety and effectiveness for new indications, the Medicines Repurposing programme commissions generic drug manufacturers to submit regulatory paperwork to the Medicines and Healthcare products Regulatory Agency (MHRA) for approval (NHS England, 2024). As a governmental healthcare payer, the NHS is motivated to support repurposing in order to improve clinical outcomes for the people it serves (National Health Service, 2021). The NHS helps drive clinical adoption of repurposed generics through their regular communications with prescribers (National Health Service, 2021).

These efforts, as well as others that facilitate drug repurposing like the European Union’s REMEDi4ALL, represent significant progress (Horizon Europe, 2022). Yet they will only be able to address a small portion of repurposing opportunities, especially in the U.S. Nonprofits like Reboot Rx and other non-manufacturers are therefore stepping in to advance more repurposed generic drugs (Naylor et al., 2015; Pantziarka et al., 2021; Weth et al., 2023; Reboot Rx, 2024). Within the current regulatory practice, non-manufacturers do not traditionally seek FDA approval for new indications, and so they are exploring other avenues, such as off-label use, to influence clinical adoption of repurposed drugs in oncology.

## Non-manufacturers can encourage adoption of repurposed drugs for off-label, standard of care use

Regulatory approval signifies a formal determination that a given drug product has been shown to be safe and effective for a specific indication. In the U.S., the FDA reviews clinical evidence in order to approve indications for an intended use in specific populations. However, the FDA does not regulate the practice of medicine, and drugs that are FDA-approved for one indication can be legally prescribed for any other use, even if it is not on the labeling of the product (“off-label use”) (Dresser and Frader, 2009; Wittich et al., 2012). Many drugs are used off-label as part of the standard of care in oncology, and such uses are often widely accepted by experts in the field (Saiyed et al., 2017; Zarkavelis et al., 2022). Off-label use decisions are influenced by oncologists, clinical practice guidelines and their committees, healthcare payers, and other stakeholders.

For off-label uses, each stakeholder group independently ascertains if there is sufficient evidence and an acceptable benefit-risk profile, which can vary by disease, stage, and availability of other treatment options.

Clinical practice guidelines are highly influential in the widespread adoption of off-label treatments into the standard of care. The guidelines are disease-specific treatment recommendations created and updated by panels of experts based on the evidence. The National Comprehensive Cancer Network (NCCN) Clinical Practice Guidelines are the most commonly used guidelines in oncology (Henry and McGivney, 2008; National Comprehensive Cancer Network, 2024a). They are referenced by oncologists when treating patients and by payers when deciding on coverage and reimbursement (National Comprehensive Cancer Network, 2024a). Many off-label treatments are included in the guidelines; only 62% of treatments in the NCCN are aligned with FDA-approved indications (Wagner et al., 2018). For example, more than half of the NCCN recommendations for metastatic breast cancer are off-label treatments (Etan et al., 2020).

Updates to the NCCN Guidelines are largely driven by an institutional review process with the NCCN member institutions (which must be National Cancer Institute-designated cancer centers). Additionally, external parties, such as pharmaceutical companies, nonprofits, and individual oncologists, can request changes to the guidelines by submitting evidence for review by the NCCN panels (National Comprehensive Cancer Network, 2024b). A small subset of the off-label treatments included in the NCCN Guidelines are FDA-approved generic drugs that were repurposed for cancer indications by non-manufacturers (Box 1). They are recognized as off-label, standard of care treatment options by the medical community. Unfortunately, there are too few examples of generic drugs gaining such recognition.

Oncologists take a personalized approach to making treatment decisions based on many factors (Msaouel et al., 2022). They determine whether off-label treatments are appropriate for their patients based on the published clinical data and their personal experiences and judgment (National Cancer Institute, 2022). They may be influenced by clinical practice guidelines, peers in the medical community, resource availability, and payer coverage decisions (Look Hong et al., 2010; American Society of Clinical Oncology, 2024; National Comprehensive Cancer Network, 2024a).

Payers, including public and private insurers, maintain drug formularies and decide which treatments they will cover or reimburse (Academy of Managed Care Pharmacy, 2019). Payers will typically cover off-label uses of drugs that either are included in major drug compendia or have evidence from one to two published clinical studies (Aetna, 1997; Centers for Medicare and Medicaid Services, 2015; Centers for Medicare and Medicaid Services, 2024; United Healthcare, 2023). In oncology, the NCCN Drugs and Biologics Compendium, derived directly from the NCCN Guidelines, is often used as part of off-label drug coverage decisions (National Comprehensive Cancer Network, 2024f).

Non-manufacturers can advance repurposed generic drugs as off-label, standard of care treatment options for cancer by engaging the key stakeholders and advocating for the use of the drugs. However, broad uptake of off-label treatments can be slow because it requires more effort from individual prescribers and patients to evaluate treatment options (Dresser and Frader, 2009; Wittich et al., 2012). When drug labeling is not kept up-to-date with new indications, patients and prescribers cannot rely on the FDA and its labeling to understand the full risk-benefit profile of drugs that could be available to them as treatment options.

Box 1 **Examples of non-manufacturers driving NCCN Guidelines inclusion for off-label uses in oncology.**

**Case study 1: Ketoconazole for prostate cancer.**

Ketoconazole (*Nizoral*) was first FDA-approved as an antifungal treatment in 1981 (Janssen Pharmaceuticals, 1981). It was initially implicated in the androgen signaling pathway after men taking the drug for fungal infections experienced the side effect of breast tissue enlargement (DeFelice et al., 1981). Following investigator-led, non-randomized Phase 2 trials, ketoconazole was added to the NCCN Guidelines in 1997 for salvage therapy in late-stage prostate cancer (Millikan and Logothetis, 1997). The first generic became available in 1999 (Janssen Pharmaceuticals, 2000). Ketoconazole’s success in treating prostate cancer paved the way for the development of abiraterone acetate (*Zytiga*), a new chemical entity with a similar biological mechanism and more favorable toxicity profile (Peer et al., 2014; The Institute of Cancer Research, 2014). Upon receiving FDA approval in 2011, abiraterone has widely replaced ketoconazole as the standard of care for metastatic castration-resistant prostate cancer (Janssen Pharmaceuticals, 2011). Ketoconazole in combination with hydrocortisone remains in the NCCN Guidelines as a secondary option (National Comprehensive Cancer Network, 2024e).

**Case study 2: Sorafenib for acute myeloid leukemia (AML).**

Sorafenib (*Nexavar*) was originally FDA-approved for the treatment of advanced renal cell carcinoma in 2005 (Bayer HealthCare Pharmaceuticals Inc, 2005; Kane et al., 2006). Sorafenib maintenance therapy has been studied for the treatment of AML since 2008, primarily by academic groups, including in two randomized controlled trials (Burchert et al., 2020; Xuan et al., 2020). In both studies, sorafenib maintenance was found to significantly reduce the risk of relapse and death in *FLT3*-ITD-positive AML patients. The first generic became available in 2020 (Mylan Pharmaceuticals Inc, 2020). Since the manufacturer did not request FDA approval or pursue inclusion in the NCCN Guidelines for AML, the Belgium-based nonprofit the Anticancer Fund submitted a request with the published clinical evidence to the NCCN (National Comprehensive Cancer Network, 2021). In 2021, the NCCN panel unanimously voted to add sorafenib maintenance for *FLT3*-ITD-positive AML to the guidelines (National Comprehensive Cancer Network, 2024c).

**Case study 3: Anastrozole for breast cancer prevention.**

Anastrozole (*Arimidex*) was originally FDA-approved for the treatment of advanced breast cancer in postmenopausal women in 1995 (Ani Pharmaceuticals, 1995). The first generic became available in 2010 (Neuner et al., 2015). The initial results of a Phase 3 randomized controlled trial published in 2013, later confirmed by long-term follow-up, found that anastrozole halved the risk of developing breast cancer in high-risk postmenopausal women (Cuzick et al., 2014; Cuzick et al., 2020). In 2018, anastrozole was added to the NCCN Guidelines for primary breast cancer prevention in postmenopausal women (National Comprehensive Cancer Network, 2024d). In 2023, commissioned by England’s Medicines Repurposing programme, a generic manufacturer of anastrozole obtained MHRA approval for prevention (Medicines and Healthcare products Regulatory Agency, 2023).

## FDA approval of new indications for generic drugs would increase utilization

Despite the ubiquity of off-label use, there are significant advantages to having FDA approval of new drug indications. FDA approval signifies a drug product has undergone the FDA’s high-caliber assessment of the safety and effectiveness data and that the benefits outweigh the risks (Kluetz et al., 2021). The translation of that data, filtered through the experience and judgment of FDA review teams, results in detailed prescribing information (i.e., drug labeling) that is the primary authoritative source for making informed treatment decisions (Price et al., 2021; Oncology Center of Excellence, 2023a). Because of this, FDA approval is valued by the medical community (Kluetz et al., 2021). Indications with FDA approval generate greater awareness, leading to a broader and more rapid impact on clinical practice patterns. Clinical practice guidelines and payers are inclined to review new FDA approvals and decide if they should be included in their guidelines and covered or reimbursed, respectively (Centers for Medicare and Medicaid Services, 2016; Academy of Managed Care Pharmacy, 2019; Linnerooth et al., 2023; National Comprehensive Cancer Network, 2024b).

Following regulatory approval, marketing campaigns can be very effective at raising awareness and increasing drug use (Ventola, 2011; Alpert et al., 2023). Pharmaceutical companies spend around $30 billion each year promoting their products directly to patients and prescribers to increase awareness and adoption (Schwartz and Woloshin, 2019). This is generally only possible for FDA-approved indications since pharmaceutical companies have significant legal restrictions in promoting off-label uses of their drug products (Van Norman, 2023).

FDA approvals are also used internationally by other regulatory agencies to inform their decisions, which accelerates and increases the global impact and patient benefit (Madhusoodanan, 2023). For example, FDA Project Orbis enables the exchange of drug labeling information and analyses to support concurrent regulatory submission and review among the FDA and participating international regulatory authorities (U.S. Food and Drug Administration, 2022c; U.S. Food and Drug Administration, 2023f).

Obtaining FDA approval for new indications is therefore important for maximizing the benefits of repurposed generic drugs for patients and ensuring accessible treatment options. A clear regulatory framework for non-manufacturers repurposing generic drugs would streamline and enhance adoption.

## Existing pathways for manufacturers to obtain FDA approval

New chemical entity small molecule drug products are first submitted for FDA review through the 505(b)(1) new drug application (NDA) pathway (U.S. Food and Drug Administration, 2016b). FDA approval of an NDA establishes a reference listed drug (RLD) based on the FDA’s findings of safety and effectiveness for the drug (Table 1) (U.S. Food and Drug Administration, 2020b). The RLD serves as the standard that other drug manufacturers may reference when seeking approval for generic versions of the drug (U.S. Food and Drug Administration, 2017; U.S. Food and Drug Administration, 2020b).

**TABLE 1:** Common terms and definitions in the U.S. FDA regulatory system (U.S. Food and Drug Administration, 2017).

Term	Definition
NDA	New drug application for FDA review and approval of a small molecule drug product that contains full reports of investigations of safety and effectiveness.
ANDA	Abbreviated NDA for FDA review and approval of a new generic drug product. The ANDA labeling must be consistent with the RLD.
Sponsor	The applicant, which assumes responsibility for the NDA or ANDA, including related clinical investigations and marketing of the drug product.
RLD	Reference listed drug from an NDA to which new generic versions are compared to demonstrate that they are bioequivalent.
RS	Reference standard designated by the FDA, typically when the RLD is discontinued, to use in bioequivalence testing and support approval of an ANDA.
sNDA	Supplemental NDA from the applicant holder for a change to their existing application.
505(b)(2) NDA	NDA pathway where at least some information the sponsor did not collect and does not have the right of reference.
Orange Book	The FDA publication “Approved Drug Products With Therapeutic Equivalence Evaluations” that contains lists of approved drug products, labeling, and therapeutic equivalence evaluations.

Sponsors must submit the following information for all NDAs for the FDA’s review: 1) clinical and nonclinical data on the safety and effectiveness of the drug for the proposed indication; 2) the proposed labeling; and 3) chemistry, manufacturing, and controls (CMC) data describing the methods of manufacturing and the controls to maintain the drug’s quality (21 CFR 314.50, 2024). Additionally, to support the processing of NDAs, sponsors incur user fees (U.S. Food and Drug Administration, 2023e).

In a 505(b)(1) NDA, the sponsor either generates all necessary clinical data or owns the right of reference to the clinical data needed to support the NDA. The 505(b)(2) NDA pathway offers a streamlined alternative to the 505(b)(1) NDA. It allows the sponsor to reference previous approvals and existing data from published literature, once any exclusivities expire (U.S. Food and Drug Administration, 1999). This can include studies not conducted by or for the sponsor, data for which the sponsor has not obtained a right of reference or use, and the FDA’s prior findings of safety and effectiveness for the RLD (U.S. Food and Drug Administration, 2019b). Over half of all NDA approvals are issued through the 505(b)(2) pathway (Premier Consulting, 2021). Most 505(b)(2) applications are for reformulated drug products or new dosage forms (Premier Consulting, 2021).

Generic drug equivalents are approved through the 505(j) abbreviated NDA (ANDA) pathway (U.S. Food and Drug Administration, 2017; U.S. Food and Drug Administration, 2019b). The purpose of an ANDA is to demonstrate that the proposed generic drug product is “the same as” an approved RLD (U.S. Food and Drug Administration, 2020b). The RLD product is usually from a 505(b)(1) or 505(b)(2) approval. Generic manufacturers must establish bioequivalence to the RLD product and submit their CMC data for their proposed drug product (21 CFR 314.94a7, 2024; 21 CFR 314.94a9, 2024). If the RLD is discontinued, the FDA will designate an ANDA to be the reference standard (RS) as a substitute for the RLD to be used in bioequivalence studies (U.S. Food and Drug Administration, 2020b). Typically, ANDAs receive a therapeutic equivalence designation in the FDA’s Orange Book, which means the drugs are considered to be interchangeable for and substitutable with the RLD (U.S. Food and Drug Administration, 2023d). ANDA holders are required to use the same labeling as the RLD, except for any differences to account for the fact that the drug products are made by different manufacturers (21 CFR 314.94a8, 2024). ANDA labeling will omit text on indications or other conditions of use that are protected by patents or exclusivities held by the RLD sponsor, provided the omission of the information does not undermine the safety or effectiveness of the generic drug for its remaining indications (U.S. Food and Drug Administration, 2023c).

Biological products are approved through the biologics license application (BLA) (21 CFR Part 601, 2024). There is currently no provision analogous to section 505(b)(2) under the law governing the approval of biological products. While the FDA is authorized to approve biosimilars, including interchangeable biosimilars akin to generics, there is no pathway that permits the addition of new indications for use to a biosimilar product short of submitting a complete, original BLA with full preclinical and clinical data. The regulatory pathways we propose are relevant for small molecule drugs and would not be applicable to biological products under current law.

NDA holders can add new indications to their existing FDA-approved labeling by filing a supplemental NDA (sNDA) (U.S. Food and Drug Administration, 2004; U.S. Food and Drug Administration, 2017). An sNDA builds on the sponsor’s data from the original NDA instead of creating an entirely new product application. The sNDA, if approved, results in updated and superseding product labeling (U.S. Food and Drug Administration, 2004). After exclusivities expire, ANDA holders can also add the new indications to their labeling through the sNDA pathway (U.S. Food and Drug Administration, 2024c).

ANDA holders have to use a more complex process to add indications to the labeling of their generic drug products if the indications are not already on the RLD labeling. Since the submission of new safety or effectiveness data is not allowed as part of ANDA submissions, a current generic manufacturer would need to present new supportive evidence for the new indication through a 505(b)(2) NDA as a supplement to their existing ANDA (21 CFR 314.54, 2024). Newly approved indications through the 505(b)(2) pathway would likely be reflected on separate drug labeling from the original ANDA products.

Sponsors that do not currently manufacture the drug could seek to introduce their own version of the drug for the new indication by filing a 505(b)(2) NDA. The sponsor would need to meet all standard requirements of an NDA, including clinical data to support the new use and establish an adequate scientific bridge to the RLD (typically a bioequivalence or comparative bioavailability bridge) (U.S. Food and Drug Administration, 2019b).

These pathways are very rarely used by pharmaceutical companies for adding indications when there are already multiple generic manufacturers of the product. As previously discussed, this is because the product will likely be subject to pharmacy-level substitution with any available therapeutically equivalent generic, even if that generic does not have the new indication on its labeling (Song and Barthold, 2018; Sacks et al., 2021; U.S. Food and Drug Administration, 2023d). The marketing exclusivities that sponsors may receive from the FDA (e.g., new drug product exclusivity) do not protect against this. Therefore, given that it may require substantial marketing efforts to ensure clinical uptake for the new use, the investment to obtain new labeling offers little to no value for manufacturers whose products have already been genericized.

## Existing pathway for non-manufacturers to obtain FDA approval, which requires the involvement of a manufacturer

We describe non-manufacturer repurposing sponsors as entities that intend to submit or reference clinical data through a 505(b)(2) NDA to expand the FDA-approved labeling of generic drugs for new indications that may already be considered the standard of care. These non-manufacturers do not produce or distribute drug products. Their intent is to show that there is substantial evidence to support the new indications through FDA approval and then advocate for their use in clinical practice.

The process for non-manufacturers to seek FDA approval would be similar to typical sponsors who contract with third-party manufacturers. Traditionally, NDA and ANDA sponsors are pharmaceutical companies or manufacturers that intend to produce and sell the drug products for which they seek approval. The FDA approval system is based on the idea that sponsors have specific identifiable physical drug products that they intend to distribute in commerce for sale and use. However, the FDA does not require that NDA sponsors directly manufacture the drug substances or the drug products, or maintain their own manufacturing facilities (ISR Reports, 2016). Some NDA sponsors operate as “virtual” entities that use contract research organizations to perform nearly all critical functions, including clinical development, drug substance and drug product manufacturing, packaging, sales and marketing, and post-marketing safety reporting and surveillance (21 CFR 312.52, 2024). Some ANDA products also rely on drug substances made by third-party contract manufacturers, who themselves are not sponsors. The third party may describe their CMC data in a drug master file and then authorize the sponsor the right of reference for FDA review (U.S. Food and Drug Administration, 2019a).

Similarly, within the current FDA statutory framework, a non-manufacturer may sponsor a 505(b)(2) NDA to obtain approval of a new indication for a generic drug by partnering with a current manufacturer of the drug - either an NDA or ANDA holder. The manufacturer would help meet the technical requirements of the 505(b)(2) application. Through this partnership, the non-manufacturer would acquire from the manufacturer:

1. *CMC data to submit with the NDA.* A non-manufacturer may acquire CMC information, or obtain a right of reference to CMC information, from a current NDA or ANDA holder to submit to the FDA with their application (U.S. Food and Drug Administration, 2019a). Any approved manufacturer of the drug product could serve as the partner and provide their CMC data directly, or provide a right of reference to their CMC data, as long as they provided the drug for the key clinical studies supporting the application or have established therapeutic equivalence to the product used in those studies.
2. *Samples to make available to the FDA if requested.* NDA sponsors need to have the drug product and other samples readily available to support the FDA review process (21 CFR 314.50e, 2024). This may include samples of the drug substance used in the drug product and reference standards and blanks. The FDA may request that the samples be sent to FDA’s laboratories for testing and validation. Since the non-manufacturer sponsor would not have a physical drug nor the capabilities to produce it upon request, they could obtain samples from the manufacturer who is providing the CMC data.

Once approved, the 505(b)(2) application would create a new drug product with indication-specific labeling, even though the drug would be identical to the existing product under the manufacturer’s previous NDA or ANDA. The 505(b)(2) application would be tied to the specific manufacturer due to the use of their CMC data, so that manufacturer would be responsible for producing and distributing the drug product for the new indication.

The new drug product for the new indication may ultimately not be introduced into the marketplace following approval. Regulations require NDA and ANDA holders to notify the FDA if a drug product is discontinued (U.S. Food and Drug Administration, 2020a). The practice of discontinuation by pharmaceutical sponsors is known and acceptable under the current FDA regulatory system and regularly occurs for a variety of reasons (Siramshetty et al., 2016). For example, the sponsor may not be prepared to market the product, the business case for the product may no longer be favorable, or the manufacturing facility may not be able to make the product to acceptable levels of quality. Discontinued drug products remain listed in the Orange Book in the “discontinued” section and in other resources, and their labeling remains referenceable unless the FDA determines that the drugs were discontinued for reasons of safety or effectiveness (U.S. Food and Drug Administration, 2022b; U.S. Food and Drug Administration, 2024a).

All sponsors, including sponsors of discontinued products, are responsible for post-marketing surveillance and adverse event reporting, provided that the NDA is active and maintained within the FDA (21 CFR 314.80, 2024). This includes annual safety reporting to the FDA with distribution data and labeling updates, as well as maintaining a toll-free number for reports of complaints and adverse events (U.S. Food and Drug Administration, 2012; U.S. Food and Drug Administration, 2016a; 21 CFR 314.81, 2024). Depending on the reasons for product discontinuation and the nature of the relationship between the manufacturer and non-manufacturer, conducting post-marketing reports may not be feasible.

Sponsors may at any time request that the FDA withdraw the NDA for a discontinued drug product. In this case, the sponsor would have to re-submit an NDA if they wanted to sell the drug in the future (21 CFR 314.161, 2024). If the FDA determines that the withdrawal was initiated voluntarily by the sponsor for reasons other than safety or effectiveness, the approved labeling would remain listed in the Orange Book (21 CFR 314.161, 2024). The findings of safety and effectiveness for the new indication, and the new labeling, would still be “FDA-approved” and referenceable, but the non-manufacturer sponsor would not be required to continue post-marketing surveillance. Manufacturers of bioequivalent drug products could add the new indication to their labeling through an sNDA after any exclusivity period, or the non-manufacturer could waive its exclusivity.

Therefore, a non-manufacturer can fulfill the technical requirements of sponsoring an NDA by partnering with a current manufacturer without ever putting a new product in commerce. One significant challenge with this approach is that third-party manufacturers may not want to provide their CMC data or drug product samples because it may prompt FDA inspection of their facilities, require an update to their CMC information, or open the door to product liability risks (U.S. Food and Drug Administration, 2022a; Maguire, 2023). We seek to address whether the same result of new approvals for repurposed generics may be achieved through a more direct pathway.

## Proposal: A labeling-only 505(b)(2) NDA for non-manufacturers to obtain FDA approval independently

As described above, the FDA regulatory framework can be applied by non-manufacturers to seek approval for additional indications of existing drug products, but the process requires cooperation and coordination with manufacturers that lack incentives to participate. It requires a large commitment of resources and the review of a product for which there may already be numerous interchangeable versions available on the market. The process is burdensome and complex, and it discourages labeling expansion.

The FDA could create a more agile mechanism for non-manufacturers to seek approval of new indications for repurposed generic drugs through an approach that is not tied to a specific drug product made by a specific manufacturer. We propose a “labeling-only” 505(b)(2) NDA as an extension of the existing 505(b)(2) approval pathway. The labeling-only 505(b)(2) would enable non-manufacturers to reference CMC information from previous FDA determinations, provide the FDA with samples of commercially available drug products, and maintain active NDAs.

**FIGURE 1:**
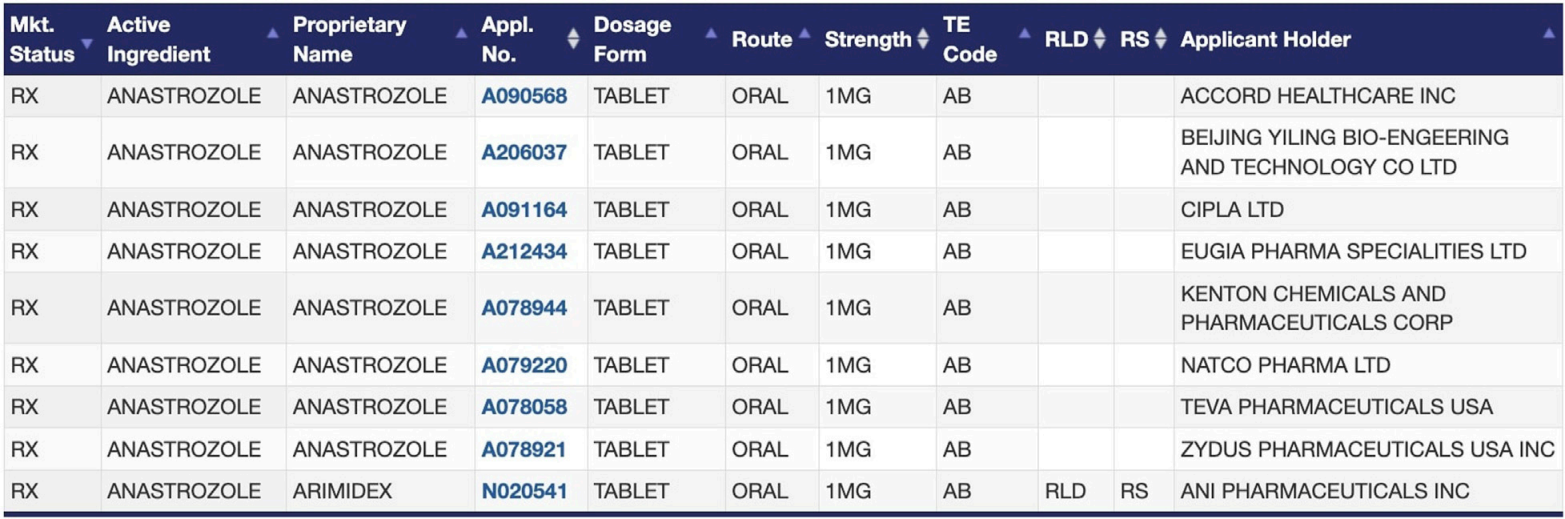
FDA-approved products for the drug anastrozole in the Orange Book, as an example of therapeutic equivalence determinations between the RLD and ANDAs (U.S. Food and Drug Administration, 2024b).

There is no inherent necessity for a new indication of a generic drug to be exclusively linked to a single manufacturer or drug product when multiple, therapeutically equivalent generic drugs have already been approved by the FDA. Any of these interchangeable drug products would be considered equally safe and effective for the new indication, and therefore there is no justification for why an indication should be exclusively linked to a specific manufacturer’s version of the drug product. The labeling-only 505(b)(2) would only be suitable for well-established, commercially available small molecule generic drugs, which can be identified as:

1. *Drugs with a U.S. Pharmacopeia and National Formulary (USP-NF) monograph.* The USP-NF monograph system, which establishes adequate consensus standards for drug substances and products, is expressly recognized in the Federal Food, Drug, and Cosmetics Act (U.S. Pharmacopeia, 2024). The goal of the USP-NF is to have substance and product monographs for all FDA-approved drugs (U.S. Pharmacopeia, 2024). USP-NF monographs for generic drugs are commonly available because the drugs have been on the market for an extended period of time and are typically produced by multiple manufacturers (U.S. Food and Drug Administration, 2019c). Drug products in the U.S. market must conform to the standards in the USP-NF, when available, to avoid possible charges of adulteration and misbranding (U.S. Pharmacopeia, 2024). The USP-NF ensures the uniformity of available products on the market by setting a consensus minimum standard of identity, strength, quality, and purity among all marketed versions of a drug (U.S. Pharmacopeia, 2019). As an illustration of the acceptance of the USP-NF, clinical trial protocols that require the use of background therapy or supportive care, as well as trials testing medical devices that require the use of a drug product, often will specify that any available version of the drug product meeting USP-NF standards can be used (Inovio Pharmaceuticals, 2017; Medtronic Corporate Technologies and New Ventures, 2019). Under our proposal, products without monographs, such as newer drugs and those with complicated manufacturing processes, would not be eligible for the labeling-only 505(b)(2) NDA pathway.
2. *Drugs with multiple A-rated, therapeutically equivalent products in the FDA Orange Book.* As the FDA does not regulate which specific drug products are prescribed, dispensed, or substituted for one another, the listing of therapeutic equivalents in the Orange Book facilitates the seamless replacement of drug products from different manufacturers in clinical practice (U.S. Food and Drug Administration, 2023d). Therapeutically equivalent drug products have demonstrated bioequivalence to the RLD, have the same strength, dosage form, and route of administration as the RLD, and are labeled for the same conditions of use as the RLD. Therapeutic equivalents that meet these criteria are designated as “A-rated” in the Orange Book. A-rated drug products are substitutable for any other version of that A-rated drug product, including the RLD itself. For example, oral anastrozole has nine versions available on the market that the FDA has determined to be therapeutically equivalent (Figure 1) (U.S. Food and Drug Administration, 2024b). Regulations generally require all versions of injectable solutions for a given drug to have nearly identical formulations, so determinations of therapeutic equivalence are even more straightforward for injectable solutions (21 CFR 320.22, 2024).

For eligible generic drugs, in their labeling-only 505(b)(2) NDA, non-manufacturer sponsors would be permitted to reference the FDA’s previous determinations that the manufacturing process and CMC data from any one of the approved NDA or ANDAs are adequate to meet regulatory standards. According to the FDA, the 505(b)(2) NDA is “intended to permit the pharmaceutical industry to rely to the greatest extent possible under law on what is already known about a drug” (Woodcock, 2014). Currently, a 505(b)(2) NDA can reference the FDA’s previous findings of safety and effectiveness for an approved drug product (U.S. Food and Drug Administration, 1999). Furthermore, the statute already allows for NDAs and ANDAs to reference the USP-NF to satisfy some CMC requirements, such as specifications of the drug substance (21 CFR 314.50d, 2024). The labeling-only 505(b)(2) NDA would build on this practice by allowing non-manufacturer sponsors to reference the full CMC requirements from the FDA’s previous determinations.

To address the need for non-manufacturer sponsors to provide drug product and other samples upon request for FDA inspection during the labeling-only 505(b)(2) NDA review, non-manufacturers could provide the FDA with samples that are commercially available from different manufacturers. It is up to the discretion of the FDA whether to request, or not request, samples in the review of an application. Given that the FDA would have already evaluated the products and their bioequivalence to the RLD during the previous reviews, it is not expected that the FDA would need to re-examine the product at the level of requesting samples, with the exception of potentially examining the packaging and physical presentation of the product for compatibility with the new indication and new conditions of use.

Under the labeling-only 505(b)(2) NDA, the non-manufacturer sponsor would not be introducing a new physical drug product into the market, but the new drug labeling with the new indication would create a reference standard. The new labeling would not inherently be associated with one specific product; rather, it would be associated with all A-rated versions of the drug product that meet USP-NF standards. The pre-existing NDA sponsor could update their labeling to add the new indication through an sNDA that references the labeling-only 505(b)(2) NDA. Regardless of whether the new indication is formally on the labeling, due to pharmacy-level substitution, patients could receive any of these drug products, thereby benefiting all current manufacturers.

Since post-marketing surveillance and adverse event reporting are drug product-specific, these would continue primarily as the responsibility of the manufacturer of the physical drug dispensed (21 CFR 314.80, 2024; 21 CFR 314.81, 2024). There would be limited post-marketing reporting required from the non-manufacturer sponsor. Non-manufacturers, therefore, may be more likely to maintain active labeling-only 505(b)(2) NDAs for the new indications.

Given that the labeling-only 505(b)(2) is intended as an interpretation of the existing 505(b)(2) NDA pathway, and existing regulations governing NDAs, it could be implemented through an FDA guidance document, rather than through notice-and-comment rulemaking. Guidance documents contain the FDA’s interpretation of the governing law, and policies pertaining to regulatory issues, including exercise of the FDA’s discretion within its scope of authority. Guidance documents often provide FDA interpretations relevant to the processing, content, and evaluation of regulatory submissions (21 CFR 10.115, 2024). The FDA could issue guidance outlining the circumstances in which the FDA may rely on previous determinations of acceptable CMC data to support a 505(b)(2) application for a new use of a generic drug for which there are multiple, approved A-rated products. It is within the FDA’s discretion to accept USP-NF monographs to meet the technical requirements of an NDA (21 CFR 314.50d, 2024). The guidance could also allow for samples of commercially available drug products to be accepted by the FDA if needed for the NDA review, and not require the other types of samples. The labeling-only 505(b)(2) NDA would eliminate undue administrative burden, enabling non-manufacturers to pursue FDA approval of new indications.

## Conclusion

Patients need new and affordable treatment options for diseases like cancer that have a devastating societal impact, and repurposing generic drugs can help address this need. Due to a lack of interest from pharmaceutical companies, nonprofits and other non-manufacturers are driving these efforts forward. Yet it is difficult for non-manufacturers to seek FDA approval, so off-label prescribing can be an effective strategy for new uses for generic drugs to be adopted into the standard of care in oncology. The number of successfully repurposed drugs is limited, in part due to this gap in the U.S. regulatory system. As momentum gains for the field of generic drug repurposing and many more new therapeutic uses for generics are discovered, we must create a mechanism for non-manufacturers to seek regulatory approval. The benefits of such a pathway would extend beyond oncology to other diseases. There have been similar calls for a simplified regulatory approval process for repurposed generic drugs in Europe (Amand-Eeckhout, 2023; van der Pol et al., 2023).

We propose policymakers implement the labeling-only 505(b)(2) NDA pathway that would allow non-manufacturers to independently obtain FDA approval for new uses of generic drugs. This would modernize the labeling process for generic drugs so that non-manufacturers can be the drivers of the updates. Patients and healthcare providers would be able to access comprehensive and up-to-date indication information on generic drugs to make informed treatment decisions. With the rigor and high standards of regulatory approval, widespread clinical adoption of repurposed drugs could be realized in a formal, predictable, and systematic manner. This would increase the utilization of low-cost and widely available generic drugs in the U.S., ultimately helping to improve patient outcomes and mitigate the financial toxicities that many patients face. A dedicated pathway for non-manufacturers would increase the availability of effective treatment options while reducing costs for patients and healthcare systems worldwide.
